# From skinner box to daily life: Sign-tracker phenotype co-segregates with impulsivity, compulsivity, and addiction tendencies in humans

**DOI:** 10.3758/s13415-022-01014-y

**Published:** 2022-06-07

**Authors:** Martino Schettino, Ilenia Ceccarelli, Mika Tarvainen, Marialuisa Martelli, Cristina Orsini, Cristina Ottaviani

**Affiliations:** 1 Department of Psychology, Sapienza University of Rome, Rome, Italy; 2 IRCCS, Santa Lucia Foundation, Rome, Italy; 3 Department of Applied Physics, University of Eastern Finland, Kuopio, Finland; 4 Department of Clinical Physiology and Nuclear Medicine, Kuopio University Hospital, Kuopio, Finland

**Keywords:** Environmental cues, Ecological momentary assessment, Autonomic nervous system, Impulsivity, Obsessive-compulsive, Addiction

## Abstract

Pavlovian conditioning holds the potential to incentivize environmental cues, leading to approach behavior toward them, even outside our awareness. Animal models suggest that this is particularly true for the so-called *sign-tracker* (ST) phenotype, which is considered to reflect a predisposition toward developing addiction-related behaviours. Despite its potential clinical relevance, few studies have demonstrated the translational validity of this model, likely due to difficulties in studying Pavlovian processes in humans. To fill this gap, we combined an ecological momentary assessment with ambulatory peripheral autonomic monitoring to test the hypothesis that traits associated with ST in preclinical studies would be associated with attribution of high incentive salience to reward-related cues. Several times for 2 days, participants were asked to rate the attractiveness of several preselected ecological rewards (e.g., coffee) and the preceding cues (the smell of coffee) while their electrocardiogram was recorded. While no absolute difference in subjective and physiological measures of motivational approach to daily cues compared with rewards emerged, individuals with high levels of impulsivity, obsessive-compulsive, and addiction-prone behaviors rated as more attractive and showed a greater increase in sympathetic arousal to cues versus rewards. The opposite pattern emerged for those with low levels in those dispositional traits, who responded more (both subjectively and physiologically) to rewards compared with their preceding cues. This study represents an attempt to answer the call to parcel complex behaviors into smaller constructs, improving the early detection of those who are vulnerable to develop psychopathological disorders, particularly in the domain of impulse control such as addiction.

**Supplementary Information:**

The online version contains supplementary material available at 10.3758/s13415-022-01014-y.

## Introduction

Environmental cues play a significant role in learning processes, serving as predictors of positive or negative outcomes, but also in motivational processes, becoming incentivized cues and eliciting approach toward them (Berridge, 2000; Bindra, 1978; Lajoie & Bindra, 1976; Rescorla, 1988). Generally, stimulus-outcome associations are extremely adaptive because they increase the likelihood of acquiring rewards that are necessary for survival (e.g., food, water, safety) and for propagation of the species (e.g., a mate); nevertheless, when cues are imbued with incentive salience, becoming desirable per se, they might promote and maintain maladaptive behaviors (Robinson et al., 2014). In these circumstances, cues can instigate pathological reward seeking in disorders determined by co-occurrence of dysfunctional inhibitory processes and strongly triggered impulses (Hofmann et al., 2009; Metcalfe & Mischel, 1999), such as in compulsive eating, gambling, hypersexuality, and drug use.

The “Sign-tracker/Goal-tracker” (ST/GT) is an animal model of individual difference of cue-reward learning, offering the possibility to investigate the individual biobehavioral vulnerability to develop addiction-related behaviors. When rodents are exposed to a Pavlovian Conditioned Approach paradigm (PCA)—in which a lever cue always precedes the delivery of a food reward—the lever cue attains predictive value (becoming a conditioned stimulus [CS]) and elicits a conditioned response; but while for GT animals the lever-cue is merely a predictor and elicits a conditioned response directed at the location of reward delivery, for ST animals the lever-cue is attributed with both predictive and incentive value, and thereby it grabs attention, attraction, and physical engagement (Flagel et al., 2009). Importantly, the divergent behaviors of ST and GT animals do not reflect differences in the ability to make cue-reward associations, but the individual variation in the attribution of incentive salience to the cue (Robinson & Flagel, 2009). That is, for ST animals, the reward’s cue attains motivational value similar to that of the reward and triggers attraction (Yager & Robinson, 2010).

Moreover, relative to GT, ST rats are more impulsive irrespective of the specific component of impulsivity, because ST animals show both higher rates of impulsive choice (Kearns et al., 2006; Tomie et al., 1998) as well as impulsive actions (Flagel et al., 2010; King et al., 2016; Lovic et al., 2011; Tomie et al., 2008). Moreover, ST animals exhibit dysfunctional checking in a model of compulsive behavior (Eagle et al., 2020; Vousden et al., 2020) and an increased vulnerability to develop addiction-like behaviors (Yager & Robinson, 2013; Robinson et al., 2014; Tunstall & Kearns, 2015). Coherently, rats expressing the ST phenotype also show deficits in top-down cognitive control originating in the medial prefrontal cortex (mPFC) (Campus et al., 2016; Campus et al., 2019), and rely on bottom-up cue salience-driven mechanisms to detect cues (Phillips & Sarter, 2020). Overall, ST vulnerability to addiction seems to be conferred by a constellation of cognitive-motivational characteristics including dysfunctional inhibitory control (Robinson et al., 2018).

Overall, animal models suggest a potential clinical value of investigating ST/GT endophenotypes in humans. In fact, ST behavior co-segregates with impulsive behavior without precisely overlapping with any of the specific sub-components of impulsivity (cognitive, motor, and nonplanning), though ST may be sharing some of the underlying neural correlates of impulsivity, such as the ventrofrontal striatal circuit (reviewed in Flagel & Robinson, 2017). Thus, the ST/GT model represents a behavioral nuance that may help linking individual differences to specific brain functioning. In line with the actual call of the Research Domain Criteria (RDoC) initiative (Kozak & Cuthbert, 2016) that exploits neurobiological scientific advances to increase our understanding of mental health, the present study has the broad aim to investigate whether sign- and goal-tracking behaviors can be found in humans, and whether this could be clinically informative.

To date, only two studies explicitly attempted to categorize the population according to the phenotypical ST/GT variation, dividing individuals based on the preferential attraction for the “sign” or the “goal” within a laboratory setting (Garofalo & Di Pellegrino, 2015; Schad et al., 2020). These studies suggest that such a distinctive pattern of salience attribution emerges also in humans, supporting the preclinical findings, albeit with important limitations (reviewed in Colaizzi et al., 2020). Most importantly, these studies are limited to laboratory contexts and not to “real life” choices, while an important attempt in the field of clinical neuroscience research is to establish whether laboratory assessment of humans biobehavioral phenotypes has external validity (Myin-Germeys et al., 2018).

In an attempt to fill this gap, the current study combined ecological momentary assessment (EMA) (Stone & Shiffman, 1994) with ambulatory physiological monitoring to evaluate whether psychological traits associated with ST/GT in the preclinical literature would predict perceived attractiveness toward rewards and the preceding cues in daily life. In a psychiatrically healthy population, we expected that individuals characterized by higher tendencies toward impulsivity, obsessive-compulsive behaviors, as well as addictive behaviors (i.e., the psychological traits usually associated with ST in preclinical studies) would be more prone to attribute incentive salience (indexed by subjective and physiological measures) to cues compared to rewards in daily life, whereas those with lower scores on those dispositional characteristics would show the opposite pattern. Among addictive behaviors, we decided to focus on problematic internet and alcohol use, because they are more likely to be observed in a sample of psychiatrically healthy young adults.

## Materials and methods

### Participants

Participants were enrolled after providing a written informed consent to a protocol approved by the Institutional Review board of the Department of Psychology (Prot. N. 547/2021). Ninety-two psychiatrically healthy individuals were recruited by word of mouth and flyers, in which they were invited to participate in a study on “individual differences on motivational processes, learning, and rewards.” The following exclusion criteria were applied: (a) a history or presence of serious medical conditions (pacemaker, cardiac arrhythmia, hypertension, diabetes, endocrine, or metabolic disorders); (b) psychiatric disorders (including drug addiction); (c) neurological disorders, including traumatic brain injury, history of childhood neurological disorders; (d) use of drugs/medications (i.e., SSRI/SNRI, sedative-hypnotic or psychotropic medications, antiseizure drugs) within 1 week prior screening; and (e) pregnancy or breast-feeding. Thirteen participants were excluded based on drugs/medications assumption and diagnosis of psychiatric disorders. Three participants were further excluded after the semi-structured interview. The final sample enrolled was comprised of 76 participants (women: *n* = 59; 77.6%) of mean age 21.4 (2.9 SD) years.

### General procedure

After signing the informed consent, participants were first asked to complete a series of online questionnaires administered via Qualtrics (https://www.qualtrics.com) to assess sociodemographic and medical information (e.g., sex, age, years of education, medical or psychiatric diagnosis, substance use). In the laboratory, participants underwent a semi-structured interview (see below for a detailed description), designed to evaluate the individual tendency to perform and perceive as rewarding a series of daily events (e.g., drinking coffee or smoking). If eligible on the basis of the semi-structured interview, they were invited to fill out a series of further questionnaires to evaluate general psychological functioning (e.g., trait anxiety), and traits that co-segregate with ST/GT (see below for a detailed description). Then, they underwent a one-on-one explanation of the EMA procedure and were instructed on how to wear the ambulatory heart rate (HR) device and remove it at night and during showers/baths. The EMA lasted 2 days in total, starting from the day after the laboratory session (see below for a detailed description). After the 2 days, participants were invited to come back to the laboratory to return the device and were fully debriefed.

### Questionnaires

The *Barratt Impulsiveness Scale* (BIS-11; Patton et al., 1995; Italian version validated by Fossati et al., 2001) is a 30-item questionnaire, designed to assess attentional (“*I don’t pay attention*”), motor (“*I act on the spur of the moment*”), and nonplanning (“*I plan for the future*”) impulsiveness. The total score was considered in the present study (Cronbach’s α = 0.70).

The *Obsessive-Compulsive Inventory Revised* (OCI-R, Foa et al., 2002; Italian version validated by Sica et al., 2009) consists of 18 items assessing six areas of obsessive-compulsive experiences over the preceding month, namely washing (“*I sometimes have to wash or clean myself simply because I feel contaminated*”), ordering (“*I need things to be arranged in a particular way*”), checking (“*I repeatedly check doors, windows, drawers, etc.*”), obsessing (“*I am upset by unpleasant thoughts that come into my mind against my will*”), neutralizing (“*I feel compelled to count while I am doing things*”), and hoarding (“*I collect things I don’t need*”). The total score was considered in the present study, with high internal consistency and reliability (α = 0.87).

The *Alcohol Use Disorders Identification Test* (AUDIT; Saunders et al., 1993) is a 10-item self-report measure developed by the World Health Organization to assess hazardous/risky alcohol consumption (“*How often during the last year have you had a feeling of guilt or remorse after drinking?*”). Internal consistency in the present study was α = 0.79.

The *Internet Addiction Test* (IAT; Young, 1998) is a 20-item self-report measure based on a 5-point Likert scale assessing the Internet usage of the subject during the past month (“*Do you feel that you stay online longer than you intend?*”). In terms of internal consistency, Cronbach’s α was 0.89 in the present study.

### Semi-structured interview

A semi-structured interview was specifically developed to evaluate an individual’s tendency to perform daily a series of prototypical reward-related events (i.e., drinking coffee), as well as the extent to which such events were perceived as rewarding. Participants were asked to think about their typical day and then, for each activity, they were asked to rate the degree to which they usually enjoyed engaging in each of these activities. Moreover, participants were asked about the timing of the day in which such events were more likely to occur. Participants were enrolled if at least three of the listed events were perceived as rewarding during a typical day. For the full semi-structured interview, please see the supplementary online material (S1).

### Ecological Momentary Assessment (EMA)

**Fig. 1:**
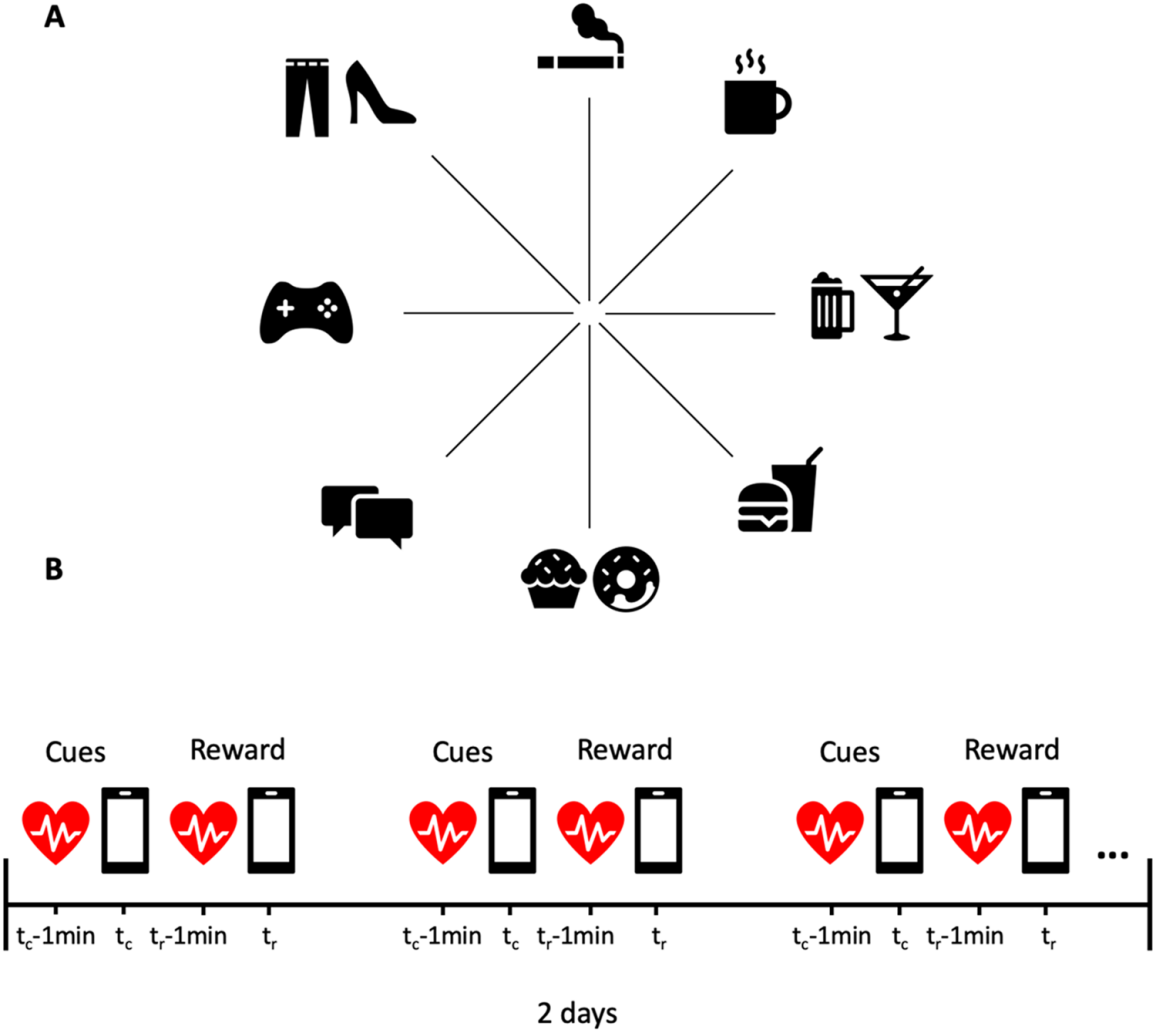
Graphical representation of the EMA procedure with a reward event-contingent design. **A** The beam chart represents the examined rewarding events. From the upper central beam to left: smoking cigarettes, drinking coffee, drinking alcohol, eating junk food, eating bakery food, using social networks, playing online games, and shopping. **B** At each reward event-contingency entry, participants are requested to fill out an anticipatory diary (t_c_) to evaluate attractiveness toward the reward-associated cues and a consummatory diary (t_r_) for evaluating attractiveness toward the corresponding reward. One minute preceding each participant’s answer was used to derive HR-event contingent for both the rewards (t_r_ -1min) and the associated cues (t_c_-1min)

To measure self-reported attractiveness toward daily occurring reward-related cues and rewards, an EMA with an event-contingent design (Myin-Germeys et al., 2018) was developed and then implemented via Qualtrics. The discrete contingent events triggering EMA assessment were predefined based on what is considered rewarding for most people. Specifically, eight typically rewarding events were considered: (a) smoking cigarettes, (b) drinking coffee, (c) drinking alcohol, (d) eating junk food, (e) eating bakery food, (f) using social networks, (g) playing online games, and (h) shopping (Fig. 1).

Reminders to fill out the electronic diaries were delivered at semi-random moments (i.e., six reminders per day every 2 hours for 2 days) and were automatically issued through an email containing two visually separated sections, each containing a list of links named after the relative reward events (e.g., coffee, shopping): the first section comprised the questions for evaluation of attractiveness toward the reward-associated cues (*anticipatory diary*); the second section comprised the questions to assess attractiveness toward the corresponding rewards (*consummatory diary*).

Each reward event-contingency entry was composed by a total of four items that were specifically developed to dissociate the response to the cue versus the anticipatory response to the reward. For the anticipatory diary, items 1 and 2 assessed the degree of self-reported attractiveness toward the perceptual cues associated with the selected rewards (e.g., for the coffee events: “*how much is the bubbling noise of the coffee machine attracting you?*” and “*how much is the smell of coffee attracting you?*”). For the consummatory diary, item 1 measured the degree of attraction toward the reward itself (e.g., “*how much are you enjoying drinking coffee?*”), and item 2 was a control item assessing whether the subject was performing two of the reward-related activities simultaneously (e.g., smoking a cigarette and drinking coffee). All items measured self-reported attractiveness on a 7-point Likert scale from 0 = Not at all to 7 = Very much.

A pilot study was conducted to make sure that 1) all the selected rewards and associated cues had the potential to elicit subjective (self-reported) and physiological (HR, rMSSD, LF/HF) attractiveness; and 2) the diary questions (“How much are you being attracted by”) were effective in detecting changes in self-reported levels of attractiveness (on a 7-point Likert scale, from 0 to 6).

The perceptual cues associated with the selected rewarding events were chosen to resemble those used in preclinical studies (e.g., sounds, visual characteristics) and for their temporal proximity to the rewards (i.e., always preceding them).

For the full set of questions, see the supplementary online material (S2).

Importantly, participants were instructed to initiate an event-contingent entry whenever they were experiencing one of the reward events with the constrain to fill out the anticipatory diary right before the reward consumption (e.g., when preparing coffee), and the consummatory diary during or right after reward consumption (e.g., while drinking coffee or immediately after).

### Physiological assessment

HR was recorded as beat-to-beat intervals (IBI) in ms with the Bodyguard 2 (Firstbeat) HR monitor. HR variability (HRV) was assessed by computing the root mean square of successive beat-to-beat interval differences (rMSSD), which reflects vagal regulation of HR and is less susceptible to respiratory influences (Penttilä et al., 2001). Sympathovagal balance was assessed using the ratio of Low- to High-Frequency HRV (LF/HF-HRV; Task Force, 1996). HR, rMSSD, and LF/HF have been used to assess implicit levels of attractiveness toward cues and rewards. The use of HRV-related measures to assess motivational approach is supported by studies finding increased HRV to substance-related cues in individuals with substance use disorders (Erblich et al., 2011, Garland et al., 2012; Ingjaldsson et al., 2003; Wang et al., 2018). Moreover, Ikisawa et al. (2020) found increases in LF/HF values as an index of approach-motivated behavior and (sexual) attractiveness.

Outlier and artifact detection as well as HRV analyses were performed using Kubios HRV software (Tarvainen et al., 2014). An automatic beat correction algorithm was used for correcting artifactual IBIs (Lipponen & Tarvainen, 2019). The beat correction algorithm detects missed and extra beat detections at 100% accuracy and ectopic beats at 97% accuracy. The detected artifacts are corrected using cubic spline interpolation. The number of 1-min segments that were corrected (beat correction > 0%) was 885, and from those that were corrected, the mean correction percent was 1.55%. Due to equipment malfunctioning and artifacts, physiological data were usable for a subsample of 13 males and 40 females.

The exact timing in which participants filled out each diary was automatically recorded on Qualtrics and the minute preceding the recorded answer was used to derive HRV-event contingent. We chose 1 minute to have adequate time for both time-domain and frequency-domain HRV analysis (Laborde et al., 2017) and to make sure that they were encountering the cues and rewards, avoiding at the same time any possible confounding effect of previous activities.

### Data analysis

Data analyses were performed with SPSS v. 27 (IBM Statistics) and PROC MIXED (SAS 9.4; SAS Institute) for general linear mixed modeling. 

First, assumptions of normal distribution, independence of residuals and sphericity were verified. We accounted for non-normally distributed scores on the OCI-R, BIS, AUDIT, IAT, IBI, rMSSD, and LF/HF values (*p* < 0.01) by performing the analyses using the natural logarithm (ln) of these measures.

Given the known sex differences in impulsivity (Cross et al., 2011), internet addiction (Su et al., 2019), obsessive compulsive tendencies (Raines et al., 2018), and HRV (Koenig & Thayer, 2016), preliminary analyses were performed to exclude the influence of sex on the main variable of interest. For the same reason, correlations between HRV-related measures and age and Body Mass Index (BMI) were performed. In these analyses, an average of HRV-related measures obtained for each minute preceding both the cues and the rewards were used. In case of significant associations, sex, age, and/or BMI were included as a covariate in the subsequent analyses.

In the analysis having HRV-related variables as outcome, we opted for not including nicotine and caffeine consumption as covariates, as the minute before anticipation (cue) and consumption (reward) diary entries were used (Hayano et al., 1990; Sondermeijer et al., 2002). Moreover, given that the anticipatory and consummatory phases regarded the same activity, there is no reason to think that physical activity could have influenced potential differences between the two.

Because the periodicity of ecological momentary measurement is likely to be highly heterogeneous, random effects regression models have been used as the most appropriate methods of analysis to test our hypotheses. All sampling moments instead of aggregated scores were used for these analyses. This procedure models each participant as a random effect, accommodating interindividual variation and dealing with missing values. Restricted Maximum Likelihood estimation was used. The covariance model among observations within subject was a random intercept plus autoregressive model.

Type of event (i.e., sign vs. reward) was related to each dependent variable: self-rated level of attractiveness, IBI, rMSSD, and LF/HF. Only the biobehavioral variables that had a significant relationship with a given dependent variable were entered. To test whether the central effects were moderated by individual differences in dispositional characteristics previously associated with sign tracking, each model was repeated by entering scores on BIS, OCI-R, IAT, and AUDIT and the moderator × Type of event interaction term as predictors. Taking into account existing arguments against the practice of dichotomizing continuous predictors (Maxwell & Delaney, 1993), questionnaires scores were treated as continuous variables in the analysis. Only to allow the interpretation of the effects of interaction on continuous variables, in case of significant moderation, a median split was used to divide participants into two groups based on low and high scores on the examined questionnaires. All the reported sign and reward events were included in all the random effects regression models.

## Results

Table 1 illustrates differences between males and females on questionnaires assessing addiction and obsessive-compulsive tendencies, trait impulsivity, on HRV-related measures, and on average self-reported levels of attractiveness. Significant sex differences emerged for IBI; therefore, sex was added as a covariate in the subsequent analyses having this variable as outcome.

**Table 1:** Sex differences in the main variables of the study

Variable	Men (*n* = 17)	Women (*n* = 59)	*t*	*p*
Age (years)	22.88 (5.17)	20.93 (1.7)	2.49	.02
BMI (Kg/m^2^)	22.38 (1.5)	22.02 (3.72)	0.34	.73
Ln(BIS)	4.03 (0.19)	4.05 (0.13)	0.73	.47
IAT	42.76 (8.63)	39.64 (11.88)	1.01	.32
Ln(AUDIT)	1.07 (0.87)	1.02 (0.83)	0.18	.85
OCI-R	2.41 (0.85)	2.51 (0.78)	0.44	.66
EMA				
Self-reported attractiveness	3.55 (1.49)	3.68 (1.75)	1.73	.08
Ln(IBI; ms)^a^	6.66 (0.16)	6.58 (0.11)	2.15	.04
Ln(RMSSD; ms)^a^	3.75 (0.46)	3.65 (0.48)	0.73	.47
Ln(LF/HF)^a^	1.66 (0.53)	1.36 (0.55)	1.71	.09

*BMI* Body Mass Index, *BIS* Barratt Impulsiveness Scale, *IAT* Internet Addiction Test, *AUDIT* Alcohol Use disorders Identification Test, *OCI-R* Obsessive Compulsive Inventory-Revised, *EMA* Ecological Momentary Assessment, *Ln(IBI)* natural logarithm of interbeat intervals, *Ln(rMSSD)* natural logarithm of root mean square of successive differences, *Ln(LF/HF)* Ratio of low- to high-frequency heart rate variability

^a^Data available only for a subsample of 13 males and 40 females

Pearson’s correlations yielded significant associations between age and IBI (*r* = 0.39; *p* = 0.004) and between BMI and *ln*(LF/HF) (*r* = 0.29; *p* = 0.03); therefore, these were included as covariates in the corresponding models.

**Fig. 2:**
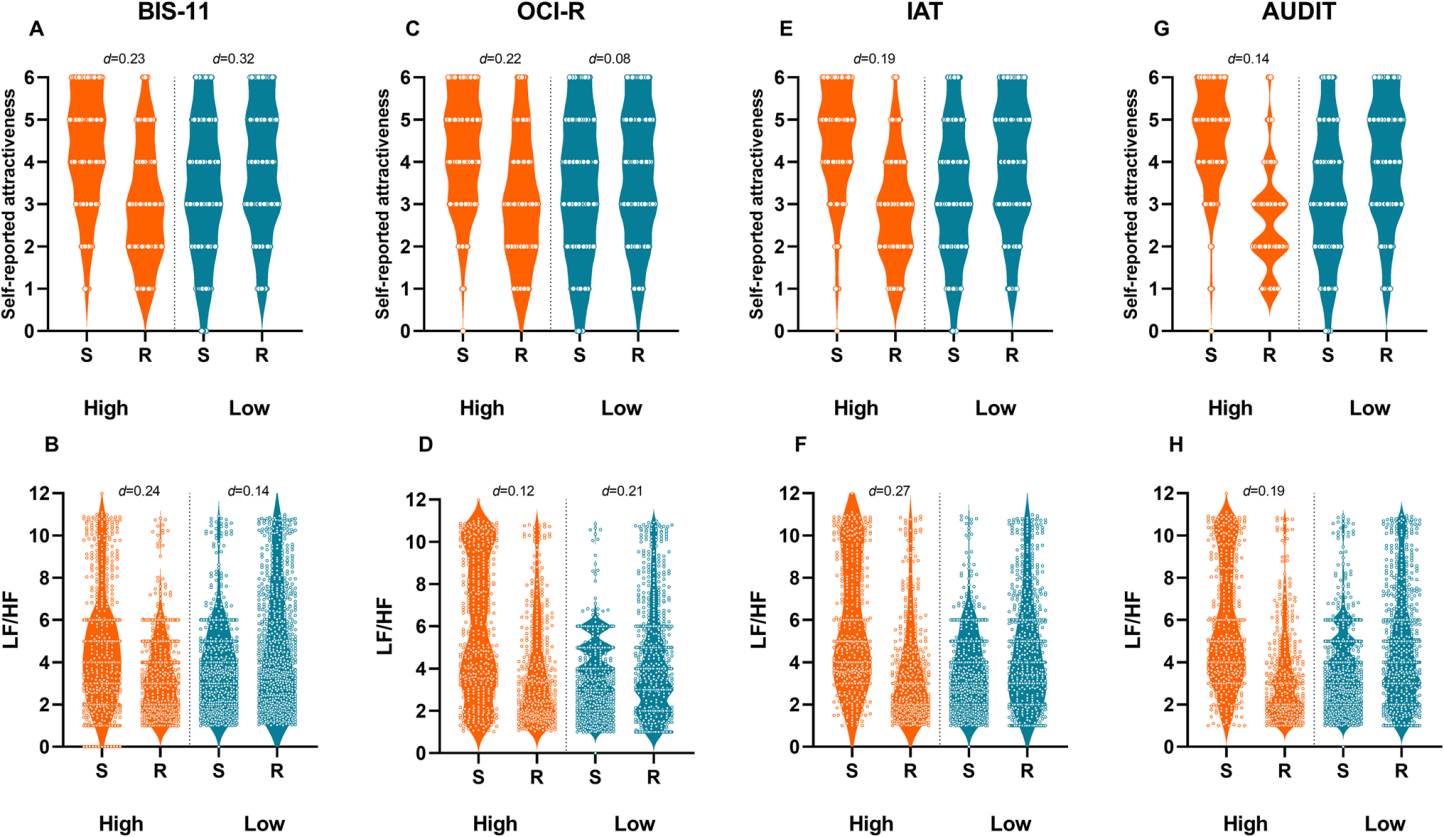
Scatter violin plot depicting each subjective (i.e., self-reported levels of attractiveness; upper panel) and objective (Low Frequency/High Frequency, LF/HF; lower panel) measures of attractiveness toward the cue and the reward for the examined groups (high- versus low-dispositional trait). Note. Each dot represents a single measurement. Cohen’s *d* effect sizes are reported for significant comparisons. For LF/HF, the statistical analyses were performed on natural log (ln) LF/HF, whereas the Figure represents nonlog data. BIS = Barratt Impulsiveness Scale; IAT = Internet Addiction Test; AUDIT = Alcohol Use disorders Identification Test; OCI-R = Obsessive Compulsive Inventory-Revised; S = sign/cue; R = reward

Random regression models showed that Type of event by itself was neither a significant predictor of momentary self-reported attractiveness nor of physiological variables. In the test for moderation effects, however, significant effects emerged. Specifically, the model having levels of momentary attractiveness as outcome, yielded significant interaction effects of type of event by all of the examined dispositional variables: BIS (*F*(11,836) = 3.85; *p* < 0.0001); OCI-R (*F*(14,833) = 3.11; *p* < 0.0001); AUDIT, *F* (7,840) = 4.62; *p* < 0.0001; and IAT, *F* (12,835) = 2.69; *p* = 0.001. To better understand these effects, we used a median split to divide participants into two groups based on low and high scores on the examined questionnaires (Fig. 2). High levels of impulsivity predicted higher attractiveness toward the sign (4.49 (1.75)) compared with the reward (4.10 (1.59), *d* = 0.23), whereas low levels of impulsivity predicted higher attractiveness towards the reward (4.56 (1.62)) compared with the sign (4.05 (1.56), *d* = 0.32) (Fig. 2A). Similarly, high levels of obsessive-compulsive tendencies predicted higher attractiveness towards the sign (4.52 (1.72)) compared to the reward (4.15 (1.62), *d* = 0.22), whereas low levels of obsessive-compulsive tendencies predicted higher attractiveness towards the reward (4.38 (1.60)) compared with the sign (4.26 (1.49), *d* = 0.08) (Fig. 2C). Moreover, high internet addiction predicted higher attractiveness toward the sign (4.50 (1.61)) compared with the reward (4.18 (1.60) *d* = 0.19), with no significant differences for low levels of internet addiction (4.34 (1.72) for the reward versus 4.30 (1.77) for the sign; *d* = 0.02) (Fig. 2E). Lastly, higher levels of alcohol use predicted higher attractiveness towards the sign (4.37 (1.67)) compared with the reward (4.15 (1.48), *d* = 0.14); with no differences for lower levels of alcohol use (4.34 (1.58) versus 4.34 (1.74), respectively) (Fig. 2G).

When physiological variables were considered as outcomes, the model having LF/HF as outcome, yielded significant interaction effect of type of event by all of the examined dispositional variables: BIS (*F*(1,1701) = 31.41; *p* < 0.0001); OCI-R (*F*(1,1706) = 36.87; *p* < 0.0001); AUDIT, *F* (*F*(1,1710) = 30.35; *p* < 0.0001); and IAT, (*F*(1,1634) = 32.84; *p* < 0.0001). More specifically, high impulsivity was associated with higher LF/HF to the sign compared to the reward (1.14 (1.04) vs. 0.89 (1.01), *d* = 0.24), whereas low impulsivity predicted higher LF/HF to the reward (1.22 (1.04) vs. 1.08 (1.01), *d* = 0.14) (Fig. 2B). High obsessive-compulsive tendencies predicted higher LF/HF to the sign (1.31 (1.03) compared with the reward (1.22 (1.03), *d* = 0.12), whereas low levels of obsessive-compulsive tendencies predicted higher LF/HF to the reward (1.07 (1.04) compared with the sign (0.84 (1.06), *d* = 0.21) (Fig. 2D). Higher internet addiction was associated with higher LF/HF to the sign (1.29 (1.02) vs. 1.00 (1.10), *d* = 0.27), whereas no significant differences emerged for low internet addiction (1.27 (1.03) versus 1.30 (1.02) for the sign and the reward, respectively; *d* = 0.04) (Fig. 2F). Similarly, higher levels of alcohol use predicted higher LF/HF to the sign (1.29 (1.02)) compared to the reward (1.09 (1.06); *d* = 0.19), whereas low alcohol use was associated with higher LF/HF to the reward (1.08 (1.05) compared with the sign (0.93 (1.08)), *d* = 0.14) (Fig. 2H). Please see the supplementary online material (S3) for the additional analyses performed to exclude any effect of habitual nicotine, caffeine, and alcohol intake on physiological responses to the specific subset of events involving (a) smoking cigarettes, (b) drinking coffee, and (c) drinking alcohol, respectively. No significant effects emerged for IBIs and rMSSD.

## Discussion

In the current study, we combined EMA with ambulatory peripheral autonomic monitoring and measures of the psychological traits found to be associated with a higher tendency to attribute motivational value to reward associated stimuli, such as impulsivity, obsessive-compulsive, and addiction-prone behaviors. We drew on preclinical evidence showing that in the ST/GT model, such psychological traits are associated with attribution of high incentive salience to reward-related cues. Current results support the translational clinical validity of sign tracking phenotype in humans.

We implemented an EMA procedure specifically developed to measure different levels of salience attribution during daily-life conditions at both explicit and implicit levels, assessed by momentary self-reported levels of attractiveness and ambulatory autonomic measures (i.e., HRV-related measures) to rewards and associated cues. Overall, participants rated cues and rewards as equally attractive. Similarly, they showed the same autonomic pattern of response to cues and rewards. This indicates that there is no absolute difference in how participants react (subjectively and physiologically) to cues versus rewards.

Notably, we found that the individual variation in the examined indicators of approach behavior emerges when psychopathological traits associated with ST in preclinical literature are considered as moderators (Eagle et al., 2020; Flagel et al., 2010; Kearns et al., 2006; King et al., 2016; Lovic et al., 2011; Robinson et al., 2014; Tomie et al., 1998, 2008; Tunstall & Kearns, 2015; Vousden et al., 2020; Yager & Robinson, 2013). In particular, individuals with higher impulsive, obsessive-compulsive, and addiction-prone tendencies (i.e., alcohol addiction and internet addiction) rated the cues (e.g., the smell of coffee) as more attractive compared to the rewards (e.g., the coffee itself). On the other hand, individuals with lower tendencies on those particular traits showed the opposite pattern, rating the reward as more attractive compared to their preceding cues. Importantly, such pattern was mirrored by autonomic measures, which pointed to increased sympathetic nervous system arousal in response to cues or to rewards, respectively in the high- versus low-dispositional trait groups.

Given that the examined patterns of salience attribution to cues and rewards are posited to result from Pavlovian conditioning, they are likely to occur outside awareness, making it difficult to study them in humans (Pool et al., 2016). For this reason, we combined subjective ratings of attractiveness towards cues and rewards with concomitant ambulatory autonomic measures, yielding convergent support to the hypothesis that dispositional difficulties in impulse control may partially overlap with ST phenotype also in humans. Importantly, we did not examine such associations in the laboratory but in participants’ daily life, using repeated assessments during multiple days and considering different types of ecological rewards (i.e., junk food, caffeine, bakery food, alcohol, nicotine, social media, shopping, videogames).

When exposed to cues preceding rewards, individuals with impulse-related difficulties (measured by levels of impulsivity, obsessive-compulsive and addiction-prone tendencies) rated them as more attractive and showed a higher sympathetic nervous system arousal (i.e., increased LF/HF-HRV), compared to that evoked by the subsequent corresponding rewards. Consistent with our results, sympathetic nervous system activation (assessed by increased skin conductance) has been previously shown to be elicited by reward-related cues following appetitive conditioning (Andreatta & Pauli, 2015; Prévost et al., 2012; Wardle et al., 2018).

Contrary to our expectations, rMSSD was not able to discriminate between different patterns of salience attribution. This seems to contradict studies showing increased vagally-mediated HRV (of which rMSSD is a measure) in response to substance-related cues in individuals with substance abuse disorders compared to controls (Erblich et al., 2011, Garland et al., 2012; Ingjaldsson et al., 2003; Wang et al., 2018). When digging further into these results, however, Ingjaldsson et al. (2003) found that among individuals with alcohol-related disorders, those with high compulsive tendencies showed reduced parasympathetic control of the heart when exposed to alcohol-related cues. This is in line with current results on reduced parasympathetic modulation in favor of sympathetic dominance (expressed by increased LF/HF-HRV) in response to cues in individuals with higher difficulties in impulse control, comprising compulsive tendencies.

The current study was not aimed at identifying humans as sign- or goal-trackers, but it represents a first ecological attempt to examine the translational clinical and predictive validity of the sign tracking phenotype as characterized by high tendency to attribute motivational value to reward associated stimuli. To date, the ST phenotype lacks clinical translation, although few previous attempts in humans exist that rely on the use of laboratory tasks. For example, Versace et al. (2016) showed that individuals with larger electrophysiological reactivity to cues predicting food delivery also reported higher scores on the attentional and non-planning impulsivity subscales of BIS-11. Albertella and colleagues found that higher attentional capture (assessed by longer reaction times) by cues signaling a reward was associated with both impulsivity and compulsivity tendencies (Albertella et al., 2019), as well as with future vulnerability to addictive behaviors (Albertella et al., 2021). Garofalo and Di Pellegrino (2015) further showed that individuals with sustained oculomotor response (an akin of approach behavior) toward cues predicting rewards were also characterized by lower impulsive control relative to those with higher oculomotor response toward reward delivery. Lastly, Schad et al. (2020) reported that sign-tracker individuals (assessed by eye-gaze direction) relied on model-free reinforcement learning rendering attractive the reward associated cue (Schultz et al., 1997).

The present results are in line with the above-mentioned reports, as individuals with high levels of impulsivity, obsessive-compulsive, and addiction-prone behaviors rated as more attractive – and showed a greater increase in sympathetic arousal to cues versus rewards, whilst an opposite pattern emerged for those with low levels of those dispositional traits. The present results add external validity, as explicit and implicit attraction were assessed outside the laboratory setting in “real” life contexts. In our opinion, the use of an EMA design represents the major strength and innovation of the current work compared to previous studies conducted on sign-tracking in humans.

The current study is not without limitations; the most important being that the sample was composed by a majority of women (77.6%). Although no differences between males and females emerged for the main variables of the study, it is well-known that females tend to have lower LF/HF (Koenig & Thayer, 2016) and lower impulse control problems (Cross et al., 2011) than males. The second main limitation concerns the explicit request to put attention to reward and cues-related information, which may have biased the subjective ratings, bringing awareness to a process that usually takes place implicitly. This might have had the consequence of increasing self-reported attractiveness and sympathetic arousal simply due to attentional orientation. However, it is implausible that the differences between self-report and sympathetic responses to cues and rewards on the basis of dispositional traits have been biased by this limitation. Third, we are unable to determine whether cues were imbued with high incentive salience in absolute terms. The present design only allowed us to infer the incentive motivational value of the cue based on self-report/physiological measures and to subsequently compare these values between individuals with high trait impulsivity, addiction, and obsessive-compulsive tendencies and those with low levels of these traits. Related to this, discrete contingent events triggering EMA assessment were predefined based on what is considered rewarding for most people. Despite our best attempt to enroll only psychiatrically healthy participants who found (at least three of) those prototypical events rewarding, we cannot exclude that analyzing each type of behavior separately (e.g., only cue/reward ratings for alcohol cues with scores on the AUDIT) would have strengthened the results.

In conclusion, assessment of the ST phenotype may represent an additional tool to make clinical predictions about individuals who are susceptible to develop psychopathological conditions. Indeed, ST is a fine-grained construct that does not fully overlap with impulsivity, which is a broad and multifaceted construct (Evenden, 1999) whose different forms are thought to depend on distinct and overlapping neural substrates (Eagle & Baunez, 2010; Dalley & Roiser, 2012). For example, the acknowledged dependence of ST phenotype on dopaminergic neurotransmission within the ventral striatum (reviewed in Flagel & Robinson, 2017) would make the identification of the neurobiological substrate of ST phenotype in humans easier compared with that underlying the complex construct of impulsivity.

The distinction between ST and impulsivity appears clear in the work of Lovic et al. (2011), in which impulsive responses accounted for about 15% of the variance of Pavlovian approach behavior, suggesting that these two traits are at least partially dissociable. To this regard, ST phenotype also is developed for cues not strictly associated to rewards, such as the safety-cues (Leclerc & Reberg, 1980), it overlaps with the construct of compulsivity both in rodents (Eagle et al., 2020; Vousden et al., 2020) and humans (Albertella et al., 2019), and it is related to a specific learning process both in rodents (Flagel et al., 2011) and humans (Schad et al., 2020).

Altogether, the present results point to the potential of ST phenotype as a transdiagnostic index of vulnerability to different psychopathological conditions. For example, it has been very recently hypothesized that the multisensorial cues associated to internet rewards (i.e., the Social Network sound-alert for a notification) may be particularly relevant for the maintenance of disordered internet use (Moretta et al., 2022). Early detection of ST phenotype may inform alternative targeted treatment program for those individuals prone to attribute incentive salience to reward-cues, who would not fully benefit from extinction-related interventions (as repeated exposure to an attractive cue can often increase attraction further).

## Conclusions

This study supports the translational clinical validity of sign tracking phenotype in humans. Our findings fit well with the actual call of the RDoC framework for an in-depth phenotypization of complex behaviors, with the goal of ultimately identifying personalized interventions and ameliorating actual diagnostic tools (Cuthbert & Insel, 2013; Insel & Cuthbert, 2015). Obviously, although the present findings are promising in a clinical perspective, replication with large-scale data and more diverse samples is warranted before causal inferences can be drawn.

## Supplementary Information

ESM 1 (DOCX 27 kb)
