# Supplementary material for: From skinner box to daily life: Sign-tracker phenotype co-segregates with impulsivity, compulsivity, and addiction tendencies in humans
Source: Cogn Affect Behav Neurosci. 2022 Jun 7;22(6):1358–69. doi: 10.3758/s13415-022-01014-y (PMC9622514; doi:10.3758/s13415-022-01014-y)
Supplement: Supplementary file 1 — (DOCX 27 kb) [file 13415_2022_1014_MOESM1_ESM.docx]

# **Supplementary Online Material**

# **From skinner box to daily life: Sign-tracker phenotype co-segregates with impulsivity, compulsivity, and addiction tendencies in humans**

Martino Schettino*^a,b*,#^*, Ilenia Ceccarelli*^a,#^*, Mika Tarvainen^c,d^. Marialuisa Martelli*^a,b^*, Cristina Orsini*^a,b^*, Cristina Ottaviani*^a,b^*

*a Department of Psychology, Sapienza University of Rome, Italy
b IRCCS, Santa Lucia Foundation, Rome, Italy*

*c Department of Applied Physics, University of Eastern Finland, Kuopio, Finland*

*d Department of Clinical Physiology and Nuclear Medicine, Kuopio University Hospital, Kuopio, Finland*

^#^ *Equal* contribution.

**S1. English translation of the full semi-structured interview**.

- At what time do you usually wake up?
- At what time do you usually go to sleep?
- At what time do you usually have breakfast?
- At what time do you usually have lunch?
- At what time do you usually have dinner?

**Coffee**

- Do you like coffee?
- In which moment(s) of the day do you usually drink coffee?
- How many coffees do you usually drink during the day?
- Among these, which coffee is the one you cannot live without?
- At what time do you usually drink this specific coffee?

**Cigarettes**

- Do you smoke?
- In which moment(s) of the day, do you usually smoke?
- How many cigarettes do you usually smoke during the day?
- Among these, which cigarette is the one you cannot live without?
- At what time do you usually smoke this specific cigarette?

**Alcohol**

- Do you like alcoholic drinks?
- In which moment(s) of the day, do you usually drink alcohol? In which occasion(s)?
- Which alcoholic drink is the one you cannot live without?
- At what time do you usually drink this specific alcoholic drink?

**Junk food**

- Do you usually eat junk food such as chips, chocolate, or fast food?
- When you want to eat something delicious that makes you feel satisfied, what do you usually eat?
- At what time do you usually eat this specific snack?

**Bakery food**

- Do you live next to a smelly bakery/pastry shop? Do you usually pass by this smelly bakery/pastry shop?
- If so, in which moment of the day/at what time do you usually step by this bakery/pastry shop?

**Social networks**

- What is your relationship with Social Networks? Do you have an Instagram/Facebook/Tik Tok account? Do you use WhatsApp?
- What is the social network that you use the most?
- Do you usually keep your phone close to you to check social networks when you are studying or working?
- In which moment of the day/what time are you usually on Social Networks?

**Game apps**

- Do you like gaming? Do you have game apps on your phone/tablet/pc?
- If so, what is the device that you use the most to play such games?
- In which moment of the day/what time do you usually play such games?

**Shopping**

- Do you like shopping? What are your favorite shops? Do you usually shop online?
- In which moment of the day/what time do you usually shop?

**S2. English translation of the full set of questions of the Ecological Momentary Assessment (EMA)**.

All the questions could be answered on the same 7-point Likert scale from 0 = Not at all to 6 = Very much.

**Coffee**

*Anticipatory diary*

- How much are you being attracted by the smell of coffee?
- How much are you being attracted by the bubbling noise of the coffee machine?

*Consummatory diary*

- How much have you been wanting to drink coffee?

**Cigarettes**

*Anticipatory diary*

- How long are you lingering on with the unlit cigarette?
- How much are you being attracted by the smell of tobacco ?

*Consummatory diary*

- How much have you been wanting to smoke your cigarette?

**Alcohol**

*Anticipatory diary*

- How much are you being attracted by the bottle of the alcoholic beverage in front of you?
- How much are you lingering on the smell of this alcoholic drink?

*Consummatory diary*

- How much have you been wanting your alcoholic drink?

**Junk food**

*Anticipatory diary*

- How much are you being attracted by the package of your chips, chocolate, or fast food?
- How much are you being attracted by the sound of the package you are opening?

*Consummatory diary*

- How much have you been wanting this snack?

**Bakery food**

*Anticipatory diary*

- How much are you being attracted by the window of the bakery/pastry shop?
- How much are you being captured by the smell coming out from the bakery/pastry shop?

*Consummatory diary*

- Did you enter the bakery/pastry shop to buy something? If so, how much have you been wanting what you have bought?

**Social networks**

*Anticipatory diary*

- How much are you attracted by your smartphone?
- How much are you attracted by the Social Network logo (e.g., WhatsApp, Facebook, Instagram) or the corresponding sound alert?

*Consummatory diary*

- How much have you been wanting to read messages or interact on social networks?

**Game apps**

*Anticipatory diary*

- How much are you attracted by the logo app or web page on which you are usually playing games?
- How much are you attracted by the sounds of the online game?

*Consummatory diary*

- How much have you been wanting to entertain yourself with gaming?

**Shopping**

*Anticipatory diary*

- How much is putting goods in your shopping cart/manipulating merchandise (e.g., trying clothes on) attracting you?
- How much are you attracted by the logo of the site/shop where you usually shop?

*Consummatory diary*

- Did you buy anything? If so, how much have you been wanting to do it?

**S3. Additional analyses controlling for habitual use of nicotine, caffeine, and alcohol**

Additional analyses were performed to exclude that the significant results obtained for the model having LF/HF as outcome were due to a conditioned physiological response to cues associated with specific rewards such as caffeine, nicotine, and alcohol, given that these rewards themselves elicit effects at a physiological level.

With this aim, additional analyses performed to exclude any effect of habitual nicotine, caffeine, and alcohol intake on physiological responses to the specific subset of events involving smoking cigarettes, (b) drinking coffee, and (c) drinking alcohol, respectively.

**Model 1:** Type of event (i.e., sign versus reward) for smoking cigarettes events only, Habitual nicontine consumption (i.e., average daily number of smoked cigarettes), and Type of event X Habitual nicotine consumption were related to LF/HF.

Results: Results: Neither Type of event (*F* (1,248) = 2.08; *p* = .150), nor its interaction with Habitual nicontine consumption (*F* (1,248) = .549; *p* = .460) were significant predictors of LF/HF. Habitual nicontine consumption significantly predicted LF/HF irrespective of Type of event (*F* (1,248) = 4.071; *p* = .045).

**Model 2:** Type of event (i.e., sign versus reward) for drinking coffee events only, Habitual caffeine consumption (i.e., average daily number of coffees), and Type of event X Habitual caffeine consumption were related to LF/HF.

Results: Results: Neither Type of event (*F* (1,255) = 0.272; *p* = .602), nor Habitual caffeine consumption (*F* (1,255) = 2.26; *p* = .134), nor their interaction (*F* (1,255) = .052; *p* = .821) were significant predictors of LF/HF.

**Model 3:** Type of event (i.e., sign versus reward) for drinking alcohol events only, Habitual alcohol consumption (i.e., average daily number of alcoholic drinks), and Type of event X Habitual alcohol consumption were related to LF/HF.

Results: Neither Type of event (*F* (1,55) = 0.45; *p* = .505), nor Habitual alcohol consumption (*F* (1,55) = .12; *p* = .732), nor their interaction (*F* (1,55) = 1.47; *p* = .231) were significant predictors of LF/HF.
